# Evaluation of students' attitude and emotions towards the sudden closure of schools during the COVID-19 pandemic: a cross-sectional study

**DOI:** 10.1186/s40359-020-00500-7

**Published:** 2020-12-17

**Authors:** Alireza Mirahmadizadeh, Keivan Ranjbar, Reza Shahriarirad, Amirhossein Erfani, Haleh Ghaem, Khoubyar Jafari, Tayebeh Rahimi

**Affiliations:** 1 Non-communicable Diseases Research Center, Shiraz University of Medical Sciences, Shiraz, Iran; 2 Thoracic and Vascular Surgery Research Center, Shiraz University of Medical Sciences, Shiraz, Iran; 3 Student Research Committee, Shiraz University of Medical Sciences, Shiraz, Iran; 4 Teenagers and youth health department of Shiraz University of Medical Sciences, Shiraz, Iran; 5 Fars Education Counselling Office, Shiraz, Iran

**Keywords:** Education, Schools, COVID-19, Emotion, Students

## Abstract

**Background:**

Rapid increases in the number of COVID-19 cases have led to the closure of academic institutions including elementary and high schools. The absence from the educational environment can affect the students’ emotions towards education and school attendance. In this study, we aimed to evaluate the students' attitude and emotions towards the sudden closure of schools during the COVID-19 pandemic.

**Methods:**

By utilizing a modified version of the Achievement Emotions Questionnaire, a web-based cross-sectional study was conducted to evaluate the students' positive and negative emotions towards schools during the early phases of the COVID-19 pandemic.

**Results:**

Questionnaires were filled by 20,697 participants with an average age of 13.76, and 70.3% of them were females. Also, 83.3% were from public schools and 80.6% from urban areas. Based on the questionnaire, for positive attitude a median of 31 (IQR 26–35) and for negative attitude a median of 25 (IQR 19–32) were obtained.

**Conclusion:**

Our findings demonstrated a satisfactory level of students' emotions regarding schools and education during the closure of schools and institutions. This indicates that despite the imposed situation, students showed enthusiasm towards learning and schools.

## Background

The rapid increase in the number of infected cases and mortalities due to the 2019 novel coronavirus diseases (COVID-19) has led to the closures of all academic institutions including elementary and high schools with the hope of slowing the transmission of the virus among the population [1, 2]. Furthermore, all students have been advised to be home quarantined regarding their safety.

A report by the United Nation Educational, Scientific and Cultural Organization (UNESCO) showed that until April 2020, school functions were affected by the COVID-19 pandemic and almost 196 countries experienced national wide closure of schools, subsequently affecting almost 1.6 billion young learners [3]. Furthermore, the Ministry of Health in Iran ordered a country wide closure of schools on March 2020 as a preventive measure in order to reduce the risk of viral transmission among the students and staff [4].

School closure in COVID-19 pandemic era has directly impacted today’s young learners. Although more than two thirds of the countries have introduced a platform for distance learning, this program was not successful in underdeveloped countries compared to developed ones with almost 30% of them being able to run a similar program. Even before the pandemic, almost 30% of world’s young population did not have access to digital educational programs, which has only gotten worse during the COVID-19 pandemic [3].

However, this is not the first incidence of school closure. During the pandemic of the H1N1 Influenza, US health officials suggested temporary school closures. A study during this period and regarding the impact of school closure on students reported that students' activities, with respect to their grades, didn’t decline during this time period; however, their interactions with other classmates remarkably decreased [5]. The absence from the academic and educational environment can affect the students’ behavior and emotions towards education and school attendance. Therefore, it can be stated that students' emotion is influenced by public health emergencies which necessitates adequate devotion and support from authorities. It is proposed that schools should collaborate in managing these situations by providing crisis-oriented psychological support and facilities for their students [6, 7].

Pekrun et al. described “emotions of progress” as emotions that are directly linked to either emotion during the activities or its consequences, which consists of various situations. Their study findings revealed that academic emotions were remarkably associated with the students’ enthusiasm, academic achievement, self-regulation, cognitive resources, and learning strategies, as well as class experiences and character [8]. Positive emotions include pride, hope, and enjoyment, while negative ones include anger, anxiety, hopelessness, shame, and boredom. The public opinion considers positive emotions to have positive consequences and negative emotions to have negative consequences; however, each of these two categories of emotions has its own benefits. Positive emotions broaden the circle of human thinking; spreading creativity, curiosity, and bonding with others; discovering social perspectives and connections; and acquiring physical and social skills. On the other hand, negative emotions are the motivational sources for self-defense, spirit of cooperation (feeling guilty), seeking justice (anger), informative aspects (for example, sadness about deficiency), and assist in learning. Negative feeling indicates a problem and, therefore, motivates us to solve that problem [8–10]. In another study, it was demonstrated that positive emotions positively predicted subsequent achievement (math test scores and end-of-the-year grades), and that achievement positively predicted these emotions, controlling for the students’ family socioeconomic status, intelligence and sex; however, negative emotions negatively predicted achievement, and achievement negatively predicted these emotions [9]. Also, Sakiz et al. state that the total effect of perceived teacher affective support on behavioral engagement was as effective as that of the students’ perceived academic self-efficacy beliefs in science [10].

Given the fact that school closure may affect the students in a variety of aspects, this study was conducted to investigate the students’ positive and negative attitudes and emotions toward the closure of schools due to the COVID-19 pandemic and to evaluate its correlation with related academic factors. Findings of this study would help to establish a well-scheduled strategy to optimize learning in the prone population.

## Methods

### Sample size

Because of the possible risks associated with the contraction of the disease, a community-based general sampling survey could not be performed; therefore, the data were collected through a cross-sectional web-based survey. The authors understand that in this method, the mentioned population would hold a selection bias and could not be a representative of the general population; however, it was the optimum approach during this critical period of time to limit the spread of the disease. Sample size estimation was calculated based on a study by Erfani et al. [11] and performed by considering a confidence level = 95%, with a d (margin of error) = 0.01, power of effect = 0.7, resulting in a calculated sample size of 8,067 participants; also, based on variations in the living locations and type of schools (private vs public) and applying a design effect of 2.5, we reached a final sample size of approximately 20,200 participants.

### Questionnaire

For the purpose of this study, the Achievement Emotions Questionnaire (AEQ) was used [12]. The questionnaire was adjusted and modified based on independent experts’ opinions in order to focus on questions and factors addressing the student's emotions. The questionnaire was subsequently pilot tested and adjustments were applied in order to enhance a better understanding of the questions by the participants before the final survey, along with looking into the arrangement of the questions for ensuring its efficiency. Eventually, a 19-item questionnaire was prepared which consisted of 8 questions regarding positive emotion and attitudes and 11 questions regarding negative emotion and attitudes, which approximately took 9 min to be filled out (Additional file 1: Table 1).

### Measures

The answers were based on the Likert Scale Score and consisted of completely agree, agree, no opinion, disagree, and completely disagree which were scored from 1 to 5, respectively. Scoring for proportional questions was reversed based on the nature of the question group (positive or negative emotion). The scores of each section were added up and evaluated among various groups. Hence, the score regarding positive emotions ranged from 8 to 40 and negative emotions from 11 to 55.

### Procedure

Our questionnaire was presented to the Vice-Chancellor for Health Affairs of the Ministry of Education who is in charge of health and hygiene and performs supervision and necessary examinations in school districts. This organization handed out the questionnaire to each school's health coach, which subsequently distributed the questionnaire though the school’s specific online family network system, established for monitoring the students during the COVID-19 pandemic school closure period. The participants could view the questions basically by entering the provided link and answering the questions. The cover page of the survey contained a brief overview of the purposes, voluntary nature of participation, procedures, and statements of confidentiality and anonymity. The inclusion criteria of our study consisted of Fars province, southern Iran students from the first grade to the 12^th^ grade (before university). As to the students who were younger or did not understand the content of the survey, we asked their parents or guardian to assist in filling out the questionnaire. Also, IP filtering was used to delete duplicate responses; the participants could withdraw anytime they wished during the survey.

The first report of COVID-19 in Iran was reported on 19th February, 2020, while the official school closure occurred on 5th of March and the nationwide quarantine was applied on 26th March, 2020. Our study was conducted during the early periods of school closure (from 14^th^ to 31^st^ of March, 2020), while the effect of this newly encountered circumstance was at its highest level. Furthermore, demographic variables including sex, age, province of residence, type of school, and Grade Point Average (GPA) were collected.

### Statistical analysis

Statistical package for social sciences (SPSS Inc., Chicago, Illinois, USA) version 26.0 was used to perform all statistical analyses. In this regard, data are presented as mean ± standard deviation (SD) or median and Interquartile range (IQR) as appropriate. Also, parametric data with normal distribution were compared between groups using an independent t-test, while those without normal distribution were compared using Mann Whitney U-test. The Likert scale was used to demonstrate the results. Pearson correlation was used to evaluate the relationship between attitude and related academic variables.

## Results

In our study, 44,993 individuals received the questionnaire, and ultimately, 20,697 filled questionnaires were received resulting in a response rate of 46%. The mean age of the participants was 13.76 ± 2.50 years which included 6139 (29.7%) male and 14,558 (70.3%) females. Of all participants, 17,238 (83.3%) were from public schools, while 3459 (16.7%) were from private schools. Among the participants, 16,672 (80.6%) were from urban areas and 4025 (19.4%) were from rural areas; furthermore, 11,305 (54.6%) were from Shiraz, the capital of Fars province and center of our study, whereas 9392 (45.4%) were from other locations in Fars province. The average grade point average (GPA) of the participants was 18.65 out of 20 (SD = 2.15). Based on the positive and negative attitude questionnaire, for positive attitude a median of 31 (IQR 26–35) and for negative attitude a median of 25 (IQR 19–32) were achieved. Table 1 shows the correlation between the positive/negative emotions of students and the variables in our study.

**Table 1:** Relationship between the study variables and positive/negative emotion

Variables	Groups	Frequency (%)	Positive emotion score median (IQR)	*p* Value	Correlation	Negative emotion score median (IQR)	*p* Value	Correlation
*Age group*								
	6–9	1594 (7.7)	34 (27, 41)	< 0.001	− 0.238	23 (13, 33)	< 0.001	0.196
	10–12	3055 (14.8)	33 (26, 40)			23 (22, 34)		
	13–15	11,379 (55)	31 (23, 39)			25 (12, 38)		
	≥ 16	4669 (22.6)	28 (20, 36)			29 (17, 41)		
*Sex*								
	Male	6139 (29.7)	31 (26, 35)	0.027	0.22	26 (20,32)	0.097	− 0.008
	Female	14,558 (70.3)	31 (26, 35)			25 (19, 33)		
*School type*								
	Public	17,238 (83.3)	31 (23, 39)	0.584	0.003	25 (12, 38)	0.114	− 0.011
	Private	3459 (16.7)	31 (22, 40)			25 (12, 38)		
*Living location*								
	Urban	16,672 (80.6)	31 (22, 40)	< 0.001	0.121	25 (12, 38)	< 0.001	− 0.064
	Rural	4025 (19.4)	33 (25, 41)			24 (13, 35)		
*Education level*								
	1–3	1752 (8.6)	33 (7)	< 0.001	− 0.226	23 (10)	< 0.001	0.179
	4—6	2330 (11.3)	33 (8)			23 (11)		
	7–9	11,798 (57)	31 (8)			25 (13)		
	10—12	4387 (21.2)	28 (8)			29 (12)		
*GPA (average from a total score of 20)*								
	≤ 15	944 (4.6)	29 (21,37)	< 0.001	0.105	30 (16, 44)	< 0.001	− 0.173
	15—18	3956 (19.1)	29 (21, 37)			29 (17, 41)		
	> 18	12,738 (86.2)	32 (23, 41)			24 (12, 36)		

## Discussion

Studies have demonstrated that public health concerns, such as the COVID-19 pandemic, may cause psychological problems for students; its presentation includes a spectrum of anger, fear, anxiety, hopelessness, and boredom [13–15]. This study aimed to investigate the positive and negative emotions of students during the COVID-19 pandemic and evaluate the elements which influence their emotions regarding school and education.

Our survey demonstrated a higher score regarding the students' positive emotions towards school compared to negative emotions (31/40 compared to 25/55, respectively). These findings reveal that students generally have enthusiasm for re-opening of school. This may be due to the time period when schools were forced to shut down, i.e. (February and March when students study for their exams and teachers are enthusiastic about education. This may subsequently indicate the student’s enthusiasm, interest, and eagerness to learn [16].

The present study showed a reverse relationship between aging and positive emotion along with a direct relationship with negative emotions. These findings are in accordance with the results of a study done by Archambault et al. that showed a decrease in both rule compliance and enthusiasm in school and determination to study during adolescence and high school periods [17].

Sex was not a significant element in the present study; however, in the literature, there was evidence in favor of sex differences in attitude towards school. Studies have reported that female students demonstrated a higher positive attitude toward school and were more eager to acquire education, contrary to male students who were less interested in school and had more negative emotions toward it [18–20].

Although our study found no meaningful relationship between positive and negative emotions and the types of schools, two studies were conducted in the Philippines to compare the motivation and attitude gap between public and private schools. They showed that positive attitude and motivation of private schools toward learning are higher than students studying in public school, which can be justified by the fact that those who study in private schools have better supports from parents and teachers which results in expediting conditions [21, 22].

Based on the results, there was a significant relationship between the location of the school (urban or rural) and emotions. Positive attitude towards schools is higher in rural areas than urban schools which is compatible with the results of a study by Swanson et al. that showed rural students had a higher tendency for graduation in comparison with urban students. The etiology of this difference can be found in, educational, socio-economic, and historical conditions [23]. Wilcox et al. described that advantages offered by small, tightly-knit communities' impact the educational system. In rural areas, while being aware of the social and economic variations affecting their communities, teachers and commissioners are working to support families to comprehend shifting educational requirements and opportunities for their children. Factors related to the improvement of education in rural areas include the potentials of academic goals, expectations, learning opportunities, strategies applied by educators to maintain and develop family relationships and involve community members, nature of individual and collective educator efficacy, and instruments for adjusting education and engaging necessary interventions for students who are at risk of dropout [24].

Verešová et al. proved that the GPA had a strong correlation with the attitude of students towards school and learning, which is parallel with our findings. Students with higher GPAs had higher positive emotions compared to negative ones toward school. This can contribute to the encouragement system provided by facilities and families that provide support and opportunities to achieve better GPAs, which in part causes a virtuous circle [25].

Our study was conducted during a specific condition and time period. The situation that was imposed by the COVID-19 pandemic made educational systems to practice distance teaching methods through online courses although the impact of this element was not evaluated in this study. A study carried out by Girija et al. on this method of teaching showed that students, especially introverts, are interested in this E-learning technique. It seems that these students prefer a warm and safe place like home for learning rather than school classes [26]. Petretto et al. suggested that providing electronic devices may not be easy for every parent during the COVID-19 pandemic, thus leading to further loss of education. Moreover, lack of internet access in some parts of the country and the need for the presence of parents for helping the younger children in usage of electronic devices may worsen this situation [27]. Also, in developing countries, these facilities may not be available and students’ education can be disturbed. Thus, it was found that further developments in E-learning and its association with students’ emotions are needed, especially in developing countries.

Although our study demonstrated a high level of positive emotions towards school, children may inadvertently miss school after having been away for some time, but these positive attitudes or enthusiasm may increase the opportunity to socialize with friends than the opportunity to learn. Another consequence of this pandemic and situation is impact on physical health of students through weight gain, especially those who live in urban areas since screen time and sedentary activities tends to rise while practicing social distancing. This survey was conducted during the early phases of the pandemic in Iran in which the educational system was not yet converted from the traditional style to online home-based education. Moreover, home-based learning during the pandemic shows how inequality affects children outside school, where some face poverty, jobless parents, and domestic violence. In some developed countries, schools also rallied together to remain open so that children who depended on the subsidized school meal program could continue to go to school and not go hungry at home, while others who lacked a conducive environment for learning at home could also go back to their schools to study.

### Limitations and strengths

Although the AEQ questionnaire is a valuable tool for evaluating the students' attitude (validity 0.77–0.93), our questionnaire was a modified and shortened version which was also translated into Persian for better understanding of our participants (Cronbach's alpha: positive attitude: 0.87, negative attitude: 0.85). It is worth mentioning that the substantial number of participants during the initial days of this public health crisis is amongst the strengths of our study although female students were over-represented. The generalizability of our findings may be limited due to a sample including mainly female students. It should also be stated that the large sample size in our survey contributes to its statistical significance; yet, it may not be the situation in practice. Future studies are suggested to be conducted to reproduce this research utilizing various overall public samples to empower the findings, and test the presence of questionnaire bias in sex invariant assembly of the modified AEQ. Also, future investigations ought to research the concurrent validity of the modified AEQ through its connection with the full-version AEQ, to test whether it could be utilized as a good assessment tool. Finally, research on the invariance of the modified AEQ questionnaire among various nations would be intriguing. In addition to the limited sample representativeness, another limitation of our study can be the web-based approach that was used for this survey. Because COVID-19 can be spread through close contacts or droplets, this approach was selected to reduce the chances of transmission; however, some bias, such as lack of internet access, remains.

## Conclusion

Most countries have temporarily closed the schools as a part of a strategy to control the COVID-19 pandemic. Our findings demonstrated a satisfactory level of the students' emotions regarding schools and education during the closure of schools and institutions. This indicates that despite the imposed situation, students showed enthusiasm towards learning and schools. However, in our study, educational programs, particularly targeting older, urban population with lower GPA scores are essential for encouraging an optimistic attitude and accelerating positive emotions in these individuals. Also, we suggest that a strategic plan should be designed to find the weak points of the educational systems, especially in students with higher negative emotions. Furthermore, designing proper frameworks for the students' education improvement and activities during a global health crisis and social distancing periods is justified.

## Supplementary information

**Additional file 1:** Questionnaire regarding the positive and negative emotions of school students during the COVID-19 pandemic school closures.

## Data Availability

Data are attached as supplementary materials, and information related to the study is in the manuscript. Please contact the corresponding author for any further data. Also, the questionnaire of this study has been added as supplementary data for further use in other studies.

## Abbreviations

AEQ: Achievement emotions questionnaire
COVID-19: The 2019 novel coronavirus diseases
GPA: Grade point average
IQR: Interquartile range
SD: Standard deviation
UNESCO: United nation educational, scientific and cultural organization
