# Supplementary material for: Evaluation of students' attitude and emotions towards the sudden closure of schools during the COVID-19 pandemic: a cross-sectional study
Source: BMC Psychol. 2020 Dec 17;8:134. doi: 10.1186/s40359-020-00500-7 (PMC7744732; doi:10.1186/s40359-020-00500-7)
Supplement: Supplementary file 1 — Additional file 1: Questionnaire regarding the positive and negative emotions of school students during the COVID-19 pandemic school closures. [file 40359_2020_500_MOESM1_ESM.docx]

**Supplementary Table 1**

Questionnaire regarding the positive and negative emotions of school students during the COVID-19 pandemic school closures

| No. | Type | The following sentences expresses your feelings during school closure due to the Corona virus disease | Completely disagree | Disagree | No opinion | Agree | Completely agree |
| --- | --- | --- | --- | --- | --- | --- | --- |
| 1 | Positive Emotions | I look forward to the reopening of schools. |  |  |  |  |  |
| 2 |  | I enjoy learning school lessons. |  |  |  |  |  |
| 3 |  | I enjoy dealing with study materials and lessons. |  |  |  |  |  |
| 4 |  | When I study, I get physically excited. |  |  |  |  |  |
| 5 |  | I have an optimistic view of studying. |  |  |  |  |  |
| 6 |  | I am optimistic that I will make good progress in my studies when schools reopen |  |  |  |  |  |
| 7 |  | I think I have the right to be happy with school closures. * |  |  |  |  |  |
| 8 |  | I'm glad that I don’t have to study * |  |  |  |  |  |
| 9 | Negative emotions | Because the amount of material is bothering and frustrating me, I don't want schools to open. |  |  |  |  |  |
| 10 |  | I get angry if I have to read a lot. |  |  |  |  |  |
| 11 |  | I am so angry when I read that I like to throw the textbook out the window |  |  |  |  |  |
| 12 |  | I get anxious when I look at the books I have to read when I start school. |  |  |  |  |  |
| 13 |  | My heart beats fast when I think of school. |  |  |  |  |  |
| 14 |  | Fear of schools not opening up scares me. * |  |  |  |  |  |
| 15 |  | I am ashamed of my own happiness in postponing my studies. * |  |  |  |  |  |
| 16 |  | I don't like going to school because when I don't know the answer to the question, I get embarrassed. |  |  |  |  |  |
| 17 |  | Because I have a lot of problems with the curriculum, I don't like schools to open. |  |  |  |  |  |
| 18 |  | School lesson and material makes me so bored that I prefer schools to be closed. |  |  |  |  |  |
| 19 |  | Studying is dull and boring. |  |  |  |  |  |
| * demonstrates reverse scoring system | | | | | | | |
